# Does High-Magnitude Centripetal Force and Abrupt Shift in Tangential Acceleration Explain High Risk of Subdural Hemorrhage?

**DOI:** 10.1089/neur.2022.0025

**Published:** 2022-07-15

**Authors:** Svein Kleiven, Xiaogai Li, Anders Eriksson, Niels Lynøe

**Affiliations:** ^1^ Division of Neuronic Engineering, Royal Institute of Technology (KTH), Huddinge, Sweden.; ^2^ Department of Community Medicine and Rehabilitation/Forensic Medicine, Umeå University, Umeå, Sweden.; ^3^ Department of Learning, Informatic, Management and Ethics, Center of Healthcare Ethics, Karolinska Institute, Stockholm, Sweden.

*Dear Editor:*

 Stray-Pedersen and colleagues^1^ present a biomechanic experiment using a surrogate model supposed to represent a 1-month-old baby (3.5 kg) exposed to violent shaking. According to the authors, previous experimental studies have focused on *impact thresholds*, and the authors claim that impact thresholds cannot be validly applied when studying *non-impact* repeated shaking. The reason for this is, according to the authors, that the loads generated depend on, for example, the elastic properties of the surrogate's surface and skull.^1^ Accordingly, the focus of the authors’ experiment was on isolated shaking, in a clinical situation often thought to be associated with isolated findings of: 1) symptoms of encephalopathy, 2) subdural hemorrhage (SDH), and 3) retinal hemorrhages. Such cases are also referred to as isolated triad findings and constitute 30–40% of all alleged shaken baby syndrome/abusive head trauma cases.^2^ Hence, focusing on shaking only and isolated triad cases, as well as the authors’ ambition with the experiment, is interesting.

When conducting these kinds of experiments, there are, however, several aspects that must be considered. The authors openly declare that only the first two shaking procedures were conducted in accordance with the original protocol. A steel wire used as a neck cable was broken, and the consequences of the broken steel wire was discussed briefly by the authors because it was suspected to have affected the subsequent acceleration measurements: “*It should be noted that during analysis it was discovered that after Volunteer 2 the measured values changed considerably. Further investigation revealed that the steel cable in the dummy neck had broken, which was assumed to have happened after Volunteer 2*.” Given that the measured values changed after Volunteer 2, the cable was most likely broken already during the experiment with Volunteer 2. And if so, it is our opinion that all the subsequent 14 shakes including those presented for Volunteers 2-15 in Table 1, and Volunteer 2 in Figure 2 and 3, should be excluded from the results.

Moreover, the accelerometer setup of the surrogate model made it possible to measure only linear accelerations. As stated by the authors in the Supplementary Material (Supplementary Table S1),^1^ the angular velocity estimates were not reliable. The authors wrote: “*Please note that, as is discussed in the main text, this approach ignores several crucial aspects and hence should not be used in practice.*” Given that Stray-Pedersen and colleagues measured only linear accelerations, only hypothetical implications for injury prediction can be discussed from those. This is obvious since SDH attributable to trauma is thought to be caused by angular kinematics, namely peak changes in angular velocities in combination with high-magnitude angular accelerations, and not by linear or translational accelerations.^3,4^ Stray-Pedersen and colleagues hypothesize that the continuous centripetal forces “*may* *cause increased pressure in the subdural compartment in the cranial roof and* *may* *cause constant compression of the brain and possibly increased stretching or shearing of the bridging veins. This* *may* *contribute to the mechanism accountable for subdural hematoma in abusive head trauma*” (“may” underlined by us).

But a centripetal loading of only 5 G is not likely to add to the stretching of the bridging veins given the nearly incompressible properties of the intracranial content.

The measurements of continuous centripetal accelerations of ∼5 G is interesting. It must be noted, however, that magnitudes of 4–10 G have been measured in many daily activities, such as hopping off a step or “plopping” into a chair or for continuous long-durations when riding roller coasters, without causing any apparent brain or vascular injury in adults and children.^5,6^

Even if we assume that the reported short duration peak linear accelerations would be a mechanism of vascular injury, the mean reported peak acceleration in the skull center of around 20 G is close to what has been reported from human volunteer experiments for sled tests of much longer durations without causing injury^7^ and much less than what is reported in non-concussive sports impacts.^8^ The authors have wisely not concluded anything from their presented results, and the Conclusion section contains only a number of speculative statements including the wording “may.” We are inclined to claim that the study by Stray-Pedersen and colleagues does not add anything substantially new. On the contrary, their own measurements question their own proposed hypotheses.

## Abbreviation Used

SDH: subdural hemorrhage
